# Development and validation of a gas chromatography–mass spectrometry method to analyze octanoate enrichments at low concentrations in human plasma

**DOI:** 10.1007/s00216-020-02801-7

**Published:** 2020-07-09

**Authors:** Dewi van Harskamp, Suzan J. G. Knottnerus, Gepke Visser, Johannes B. van Goudoever, Henk Schierbeek

**Affiliations:** 1 Amsterdam UMC, Stable Isotope Research Laboratory, Endocrinology, Amsterdam Gastroenterology Endocrinology and Metabolism, University of Amsterdam, Meibergdreef 9, 1105 AZ Amsterdam, The Netherlands; 2 Amsterdam UMC, Emma’s Children’s Hospital, Amsterdam Gastroenterology Endocrinology and Metabolism, University of Amsterdam, Meibergdreef 9, 1105 AZ Amsterdam, The Netherlands; 3 Department of Metabolic Diseases, Wilhelmina Children’s Hospital, University Medical Center Utrecht, Lundlaan 6, 3584 EA Utrecht, The Netherlands; 4 Amsterdam UMC, Laboratory Genetic Metabolic Diseases, Amsterdam Cardiovascular Sciences, Amsterdam Gastroenterology Endocrinology and Metabolism, University of Amsterdam, Meibergdreef 9, 1105 AZ Amsterdam, The Netherlands

**Keywords:** Stable isotopes, Mass spectrometry, Medium-chain triglycerides, Elongation

## Abstract

A new method for accurately analyzing octanoate enrichment in plasma was developed and validated. Samples were derivatized directly in plasma by transesterification with isobutanol and were analyzed by gas chromatography–mass spectrometry (GC-MS). This method was developed to analyze the precursor enrichment in a stable isotope tracer protocol. Glyceryl tri[1,2,3,4-^13^C_4_] octanoate, a stable isotope-labeled medium-chain triglyceride (MCT), was orally administered in combination with (1) exclusively MCT or (2) a combination of protein, carbohydrates, and MCT to investigate the metabolic route of oral MCT under various conditions. Accurate analysis of octanoate enrichment in plasma at concentrations as low as 0.43 μM (lower limit of quantification, LLOQ) was performed. This is an improvement of about twenty times for the LLOQ for analysis of the enrichment of octanoate when compared with the gold-standard method for fatty acid analysis (methyl esterification). Moreover, we found that‚ with this gold-standard method, study samples were easily contaminated with (unlabeled) octanoate from other sources, leading to biased, incorrect results. The precision and linearity obtained using the new method were good (coefficient of variation intraday < 9.1%, interday < 9.3%, *R*^2^ of the calibration curve > 0.99). The sensitivity was sufficient for analyzing samples obtained using the stable isotope protocol. This new method is more sensitive than methyl esterification and it minimizes the risk of contamination.

**Figure:**
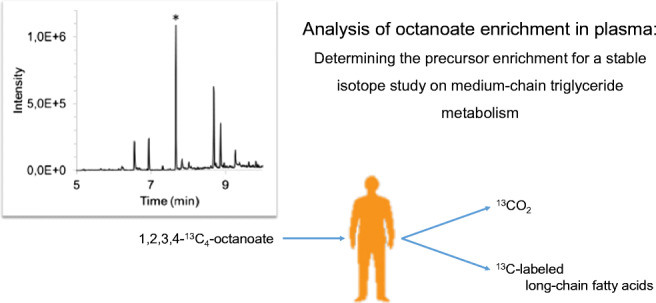
Graphical abstract

Graphical abstract

## Introduction

One of the clinical applications of medium-chain triglycerides (MCT) is as a dietary supplement for patients with a disorder involving mitochondrial long-chain fatty acid oxidation [1]. These patients are unable to oxidize long-chain triglycerides (LCT), but are capable of oxidation of MCT. Although both are fats, MCT and LCT are processed differently within the body. For example, their routes of oxidation vary. Fatty acids from LCT have a chain length of 14–18 carbon atoms and need enzymes to enter the mitochondria, where they can be oxidized. In contrast, medium-chain fatty acids, which have a chain length of 6–12 carbon atoms, can diffuse passively through the mitochondrial membranes [2]. MCT oxidation circumvents per definition the defective pathway of the patients with a disorder involving mitochondrial long-chain fatty acid oxidation. In this way, MCT is able to compensate for the insufficient long-chain fatty acid oxidation and serves as an alternative energy source to patients with an impaired long-chain fatty acid oxidation.

However, the exact utilization of MCT when administered as a dietary supplement in humans is largely unknown. There is evidence of MCT elongation in premature infants [3] and of its use as an energy source when it is consumed before exercise [4]. Current research in our team is geared towards understanding the metabolic fate of MCT after supplementation, in combination with different feeding regimes. To this end, we proposed a stable isotope protocol in which we analyze the utilization of an orally administered stable isotope–labeled MCT (glyceryl tri[1,2,3,4-^13^C_4_]octanoate) in vivo under different nutritional regimes. The use of stable isotopes in clinical studies is a safe way to trace and calculate the contributions of pathways within metabolism that otherwise cannot possibly be assessed [5]. With this stable isotope protocol, where the tracer is supplied orally via a primed continuous administration, we will have a unique insight in the in vivo utilization of dietary MCT and will gain knowledge on the relative direction of metabolism towards oxidation or elongation of the dietary MCT. As the tracer infusion is combined with different supplementations (i.e., MCT only, or MCT combined with protein and carbohydrates), the influence of these supplements on the relative direction of metabolism can be assessed. The stable isotope protocol, once validated, is to be applied to adult patients with a long-chain fatty acid disorder, to determine whether elongation contributes significantly to MCT metabolism, which is unfavorable for this patient group. Additionally, knowledge of the influence of the additional supplemental nutrients to MCT metabolism may lead to a reconsideration of the current advice to patients on how to administer the MCT supplement.

^13^C-labeled octanoate is frequently used for breath testing (e.g., [6, 7]), to determine its oxidation or as a marker for gastric emptying (e.g., [8, 9]). In these protocols, there is no need to analyze the precursor enrichment in plasma, and ^13^CO_2_ enrichment is determined in breath. ^13^C-octanoate has previously been used for elongation studies and analyzed by methyl esterification [3]. Methyl esterification of fatty acids followed by gas chromatography–mass spectrometry (GC-MS) or gas chromatography–combustion–isotope ratio mass spectrometry (GC-C-IRMS) analysis is considered the gold standard, both for concentration and for enrichment analysis [10, 11]. Unfortunately, in samples obtained with the proposed isotope infusion protocol, the concentration of octanoate was below the limit of detection for GC-C-IRMS analysis, and with GC-MS analyses, we experienced discrepancies when analyzing the octanoate methyl ester. Therefore, a new method was proposed.

To measure the precursor enrichment in plasma samples obtained during the stable isotope protocol, we aimed to develop and validate a GC-MS method to determine the enrichment of the 1,2,3,4-^13^C_4_-octanoate tracer at low concentrations in plasma.

Here, we present this optimized new method, which is based on derivatization by transesterification with isobutanol. With this method, the total plasma octanoate enrichment is analyzed. We altered our current methylation protocol [12, 13] to facilitate isobutylation, which in comparison with methylation reduced the volatility of the derivatized octanoate, thereby reducing the risk of losses of the analyte during sample preparation. Direct derivatization in plasma limits the risk of contamination and, therefore, has improved accuracy and reproducibility. To our knowledge, this is the first description of this derivatization procedure used for the enrichment analysis of octanoate in human plasma.

## Materials and methods

### Reagents

Isobutanol (EMSURE grade; Merck, Darmstadt, Germany), acetyl chloride (Sigma, Schnelldorf, Germany), chloroform (SupraSolv grade; Merck, Darmstadt, Germany), hexane (Merck, Darmstadt, Germany), octanoate (Sigma, Schnelldorf, Germany), and Liquigen^®^ (Nutricia, Zoetermeer, The Netherlands, containing a.o. 27 g/100 mL glyceryltrioctanoate and 18 g/100 mL glyceryltridecanoate) and glyceryl tri[1,2,3,4-^13^C_4_] octanoate (Sigma, Zwijndrecht, The Netherlands) was prepared by the pharmacy for oral administration during the study protocol. It consisted of the tracer in a concentration of 0.85 mg/mL with methylhydroxybenzoate as a preservative. The prepared solution was homogeneous enough for the oral infusion, though pipetting small amounts (e.g., 20 μL) turned out to be problematic. Therefore, a 0.5 mL portion was extracted by 4 mL chloroform, 2 mL methanol, and 0.8 mL 0.9% NaCl and vortexed for 1 min. This was centrifuged for 10 min at 2500*g*. The lower layer was transferred into a clean tube, and the liquid–liquid extraction was repeated with 4 mL of chloroform. The lower layer was combined with the portion from the first extraction step, and the combined layers were dried under a gentle flow of nitrogen. The residue was reconstituted in 500 μL hexane. This solution was tested for its concentration and isotopic purity and was used for preparation of the calibration curve and control samples.

### Calibration curve

To establish a calibration curve for enrichments, varying amounts of the extracted labeled octanoate in hexane solution were added to a fixed amount of unlabeled octanoate (64 nmol/mL), resulting in calibration standards with known enrichments. The enrichment levels were 0.0, 3.5, 13.8, 20.7, 27.7, 55.3, and 83.0 tracer-tracee ratio percent (TTR%, the amount of tracer divided by the amount of tracee, multiplied by 100). After analysis, the peak area for the labeled octanoate was divided by the peak area for the unlabeled octanoate (experimental tracer-tracee ratio [TTR]). These results were plotted against their known enrichments, expressed as TTR%. The slope and intercept of this graph were used to correct the experimental results of the samples.

### Control samples

Pool plasma was spiked with labeled octanoate to create control samples at two different enrichment levels. To increase the endogenous unlabeled octanoate level to a relevant concentration, a combination of labeled and unlabeled octanoate was added to the pool plasma (low control: 150 μL of a 0.21 μmol/mL 13.8 TTR% enriched octanoate solution per mL of pooled plasma; high control: 200 μL of a 0.16 μmol/mL 27.7 TTR% enriched octanoate solution per mL of pooled plasma). As endogenously present unlabeled octanoate present in plasma dilutes the enrichment added during the spiking of the controls, the resulting enrichments were 4–11% TTR%. As the stock solution of glyceryl tri[1,2,3,4-^13^C_4_] octanoate was dissolved in an organic solvent, the spiking solution was carefully dried under a gentle flow of nitrogen. Afterward, pool plasma was added. If the stock solution and plasma were mixed directly, a phase separation was apparent and it was impossible to obtain homogeneous control samples, leading to a large imprecision of the enrichment analysis results. With the current preparation procedure, this was not observed.

### Isobutanol derivatization

Prior to sample pretreatment, every piece of glasswork was rinsed thoroughly with chloroform and baked for at least 2 h at 80 °C to prevent the contamination of octanoate from the glasswork. For the analysis of total octanoate enrichment in plasma, the following sample preparation steps were performed: in a GC vial, 100 μL of either plasma, the control sample, or the calibration standard was mixed with 200 μL 0.213/1 (v/v) acetyl chloride in isobutanol, resulting in a concentration of 3 mol/L. This reagent was freshly prepared for every experiment. Acetyl chloride should be added slowly during the stirring of the solution; as the mixing of these reagents is very exothermic, the reagents can be cooled prior to mixing to reduce the exothermic reaction. The samples were then incubated for 60 min at 90 °C. After the samples cooled to room temperature, 250 μL chloroform was added to them; they were vortexed for 1 min and centrifuged for 5 min at 1500×*g*. A portion of the lower phase was then transferred to an insert in a GC vial.

### Analysis

A 7890A gas chromatograph (GC), equipped with a 7693 autosampler coupled to a 7000 triple quadrupole mass spectrometer (MS/MS) (Agilent Technologies, Amstelveen, The Netherlands) (GC-MS/MS), was used to perform the analysis. The GC was equipped with a Dean’s switch to reduce contamination of the source through the sending of the effluent prior to and after elution of the octanoate peak to waste. Samples were introduced using a temperature-programmed multi-mode inlet. The GC was fitted with a VF17ms column (30 m, 0.25 mm, 0.25 μm). Helium was used as a carrier gas at a flow rate of 1.2 mL/min. Aliquots of 1 μL of sample were injected and each sample was analyzed in triplicate. The initial oven temperature was kept at 55 °C for 1 min. Then, the temperature was increased by 20 °C/min to 130 °C and held constant for 2 min. It was then increased by 5 °C/min to 160 °C followed by an increase in temperature of 30 °C/min to 300 °C; there, it was held constant for 5 min. The run time was 22.4 min. The transfer line temperature was set to 280 °C. The ion source installed was an electron ionization extractor ion source, operated at 230 °C and 70 eV. The effluent was recorded in single ion monitoring (SIM) mode from 5 to 22.4 min. The ions with an *m*/*z* value 127.1 (unlabeled octanoate, or M) and *m*/*z* value 131.1 (1,2,3,4-^13^C_4_-octanoate, or M+4) were analyzed in a more narrow resolution (0.45 amu at half-height) than the default setting (0.7 amu at half-height) to prevent a contribution from the neighboring *m*/*z* value to the *m*/*z* value of interest. The ions were recorded with a gain factor of 10 and a dwell time of 50 ms. The acquisition software to operate the GC-MS/MS was Mass Hunter GC/MS Acquisition B.07.06.2704 (Agilent Technologies, Amstelveen, The Netherlands).

### Method validation

The precision was established through repeated analysis of the control samples at different enrichment levels (4–11 TTR%). The interday precision was determined through analysis of two replicates (including preparation and triplicate analysis) of each of the control samples (at a lower enrichment and a higher enrichment) on five consecutive days. The intraday precision was determined through analysis of seven replicates (including preparation and triplicate analysis) on a single day at both a lower enrichment level and a higher enrichment level. We prespecified the maximum allowable coefficient of variation (CV) as < 10% for intraday precision and CV < 15% for interday precision.

Linearity was determined through repeated analysis of the calibration curves. The *R*^2^ value of the regression analysis over the linear range of this should be > 0.99, while the slope of this curve should be 0.01 ± 20%, which confirms that the experimental TTR (peak area tracer/peak area tracee) agrees with the theoretical value (ratio tracer/tracee present in calibration standards, expressed in TTR%). Theoretically, upon plotting of observed data against their known units, the slope is 1 if there is total agreement between the observed and theoretical values. In our case, by simultaneous interconversion to the unit used to express the enrichment (TTR% as opposed to TTR), the expected slope is 0.01. By definition, the upper and lower limits of quantification were determined and defined by the domain of linearity of the enrichment curve and by the linear dynamic range of the instrument. The minimum signal should be > 10 S/N, which is the lower limit of quantification.

The accuracy was determined from analysis of calibration standards. As there is virtually no natural background enrichment on the M+4 signal (theoretically 0.0045%), the uncorrected ratio of the MS signal should be corresponding to the theoretical ratio.

### Data analysis

Integration of the peak area was performed using Mass Hunter Quantitative Analysis software version B.08.00 (Agilent Technologies, Amstelveen, The Netherlands). Further calculations were performed using Excel 2016 (Microsoft Corporation, Redmond, USA). The slope and intercept of the enrichment curves (experimental TTR vs theoretical TTR) were determined by linear regression analysis.

### Tracer and supplement administration protocol

The study protocol was approved by the Medical Ethical Committee of the Amsterdam University Medical Center (NL59693.018.16). All procedures followed were in accordance with the ethical standards of the responsible committee on human experimentation (institutional and national) and with the Helsinki Declaration of 1975. Informed consent was obtained from all participants before their enrolment in the study. Three participants were included in this pilot study.

**Fig. 1:**
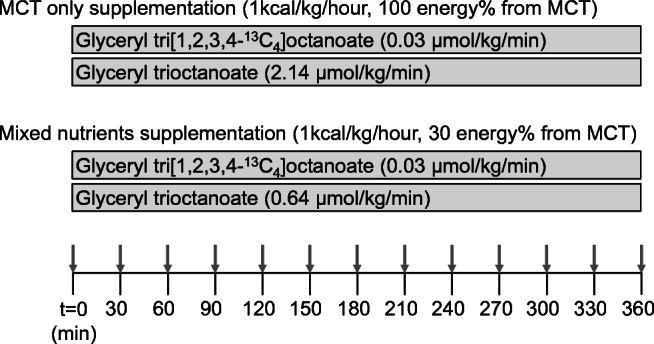
Schematic representation of the administration of the tracer and the unlabeled glyceryl trioctanoate. Both supplementations were isocaloric but differed in composition (100 vs 30 energy% from MCT). In this example, the tracer-tracee ratio percent (TTR%) of the glyceryl trioctanoate administered was 1.5 TTR% for the MCT-only supplementation and 5.1 TTR% for the mixed nutrient supplementation. The timeline below indicates the duration of the protocol (360 min) and the arrows indicate blood sampling

The protocol involved the oral administration of glyceryl tri[1,2,3,4-^13^C_4_] octanoate on two separate test days. After an overnight fast, the healthy male volunteers received a primed continuous infusion of tracer per nasogastric tube, in combination with an isocaloric feeding consisting of either MCT only, or MCT, protein, and carbohydrates. The administered amount of tracer was kept constant on both days; hence, the enrichment of the administered feeding differed as a result of the dilution by the distinct amounts of unlabeled MCT supplied during either feeding. Blood samples were withdrawn at regular intervals. An example of the administration protocol is provided in Fig. 1.

## Results

**Fig. 2:**
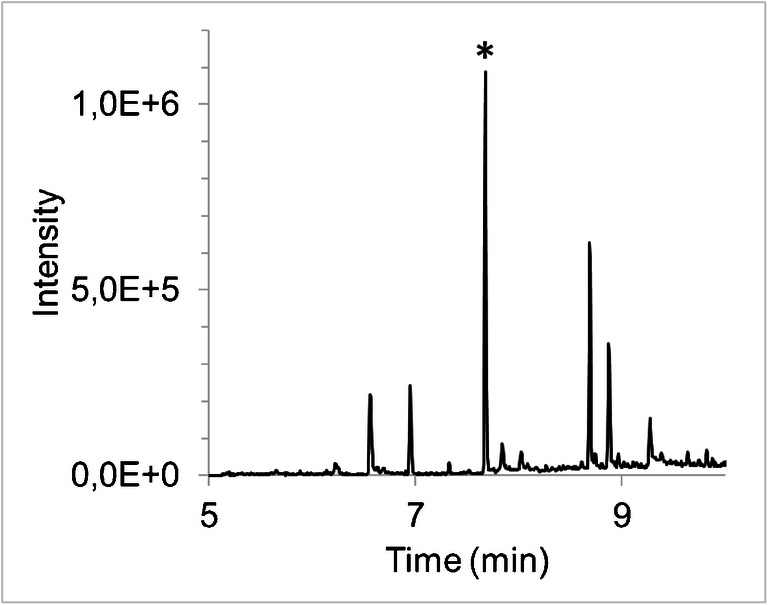
Chromatogram of pool plasma acquired in the single ion monitoring (SIM) mode for *m*/*z* value 127.1. The octanoate peak is indicated with an asterisk (*). One of the components in the chromatogram (RT 8.9 min) was identified with a high probability as the isobutyl derivate of fumaric acid. The others could not be identified through the NIST library

**Fig. 3:**
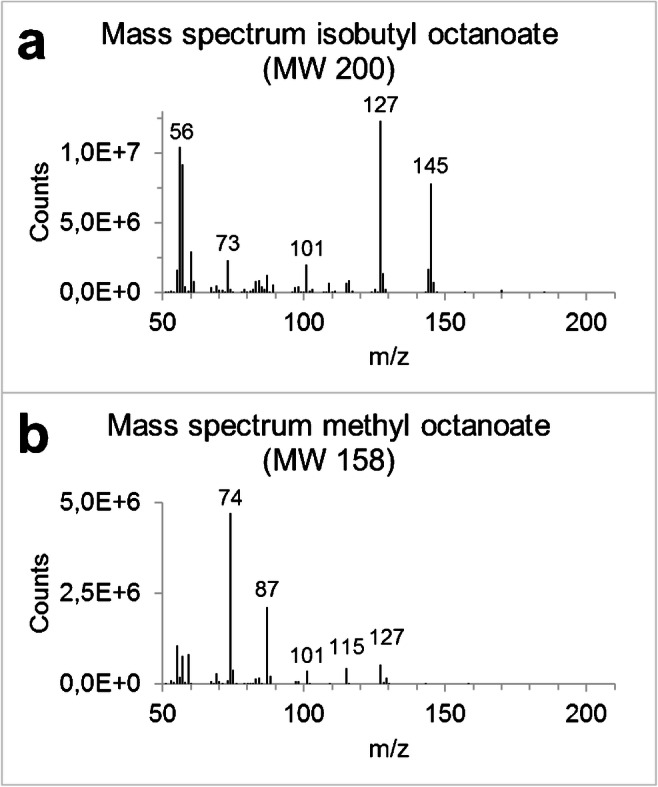
Mass spectra. **a** Obtained after derivatization of octanoate by isobutylation. Mass fragment 145 results from a McLafferty + 1 rearrangement (M-55, loss of C_4_H_7_), while mass fragment 127 results from loss of the derivate group C_4_H_9_O (M-73). In these fragments, all isotope-labeled positions of 1,2,3,4-^13^C_4_-octanoate are preserved. **b** Obtained after the derivatization of octanoate by methylation

We developed a new gas chromatography–mass spectrometry (GC-MS) method for plasma octanoate enrichment analysis using isobutylation. A chromatogram and a mass spectrum are presented in Figs. 2 and 3a, respectively. As can be seen from Fig. 2, the octanoate isobutyl ester peak was well separated from other peaks observed in this extracted ion chromatogram. The chromatogram was obtained during the analysis of pool plasma (pooled from plasma samples obtained from several patients). Therefore, this is a representative plasma sample, as potential individual differences are averaged out.

The procedure was highly similar to the gold-standard method for fatty acid enrichment analysis, where fatty acids are extracted, derivatized by addition of 3 M methanolic HCl, and incubated for 60 min at 90 °C [12, 13]. Except for the extraction and derivatizing reagent, these reaction conditions were kept the same. Experiments were conducted to check the capability of hydrolysis and transesterification of this derivatizing reagent by derivatization of glyceryltrioctanoate standards (full recovery was observed). Further experiments were conducted to find the optimal reagent volume to derivatize plasma samples, compromising between reaction recovery and dilution of the response on GC-MS (data not shown). Unfortunately, isobutanol is highly miscible; therefore, liquid–liquid extraction after derivatization was not possible, and a large excess reagent volume leads to an unnecessary dilution of the analytes. To improve the phase separation of the water layer (from plasma) and the organic layer (from the derivatizing reagent), different solvents were tested, including chloroform, dichloromethane, and water. The addition of chloroform resulted in an improved separation, did not affect the results, and was compatible with GC-MS injection.

For SIM analysis, the spectrum obtained for octanoate isobutyl ester is more favorable than the spectrum obtained for octanoate methyl ester (Fig. 3b). Both spectra were recorded by analysis of an octanoate standard, where a 20 μL aliquot of a 0.03 μmol/mL solution in chloroform was derivatized for either of the derivates. More specific mass fragments (at higher *m*/*z* values) are present in the octanoate isobutyl ester spectrum (at *m*/*z* 127.1 and 145.1 for the unlabeled octanoate isobutyl ester), which will lead to more specificity in the GC-MS method after isobutylation compared with methylation. Moreover, these acquired mass fragments of the isobutyl ester are significantly more abundant than the higher *m*/*z* mass fragments of the octanoate methyl ester, as can be seen from a comparison of the mass spectra in Fig. 3. This higher abundance of the mass fragment that will be used in SIM analysis results in a higher sensitivity. The lower limit of quantification (LLOQ) for enrichment analysis of octanoate isobutyl ester was 0.43 μM. This was an improvement of about twenty times when compared with methyl esterification. With methyl esterification, the sensitivity was too low for analysis of the isotopic enrichment of labeled octanoate in the samples obtained during the stable isotope protocol, while it was sufficient with the isobutyl ester. The extracted ion chromatogram obtained with *m*/*z* value 145.1 showed minor disturbances in the baseline of the chromatogram, affecting the accuracy of the method. Therefore, *m*/*z* values 127.1 and 131.1 were chosen for octanoate and 1,2,3,4-^13^C_4_-octanoate, respectively. Extracted ion chromatograms obtained for these ions were free of interferences in the baseline.

Reproducibility was assessed through repeated analysis of spiked control samples at two levels. We predefined the maximum allowable intraday and interday precision criteria, as described in the “Materials and methods” section. For the control sample at the lower enrichment level, the interday variation was 9.3% (coefficient of variation, CV) and the intraday variation was 4.7% CV. For the control sample at the higher enrichment level, the interday variation was 9.2% CV and the intraday variation was 9.1% CV.

**Fig. 4:**
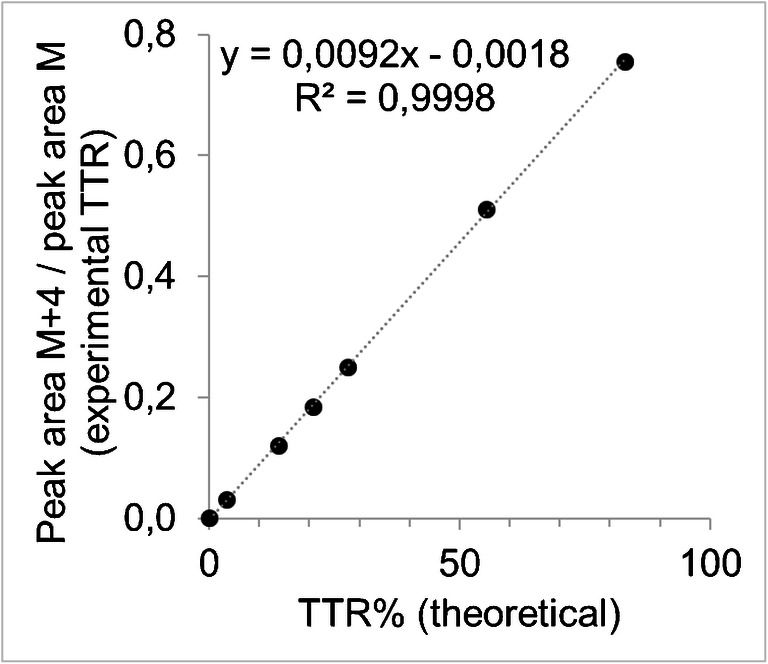
Calibration curve for the enrichment of 1,2,3,4-^13^C_4_-octanoate. The peak area for 1,2,3,4-^13^C_4_-octanoate was divided by the peak area for unlabeled octanoate for the calibration standards to obtain the experimental tracer-tracee ratio (TTR). These results are plotted against the theoretical TTR% (amount of tracer, divided by the amount of tracee, multiplied by 100) of the standards. The theoretical value of the slope is 0.01

The linearity was good during the experiments, with an average slope of 0.0091 (determined from 5 replicates, interday variation was 2.4% CV). This confirms the agreement of the experimental TTR with the theoretical value expressed as TTR%. Also, the *R*^2^ value was > 0.99 during all experiments. A calibration curve obtained during validation of this method is shown in Fig. 4.

The accuracy was determined from comparison of the raw data for calibration standards with their theoretical TTR. The average deviation of the TTR of the raw results (without correction of the calibration curve) was − 9.7% (ranging from − 7.5 to − 12.2%). After correction for the slope and intercept, the deviations of the results for the individual standards from their theoretical value ranged from − 2.7 to 5.1%.

Liquigen^®^ (Nutricia, Zoetermeer, The Netherlands) is an MCT emulsion and was used as the MCT supplement in the infusion protocol. The concentration of octanoate in Liquigen^®^ and the purity of the glyceryl tri[1,2,3,4-^13^C_4_] octanoate (Sigma, Zwijndrecht, The Netherlands) solution prepared by the pharmacy were determined using this method. For the determination of the concentration of octanoate, to an aliquot of Liquigen^®^, a precisely known amount of the tracer solution was added. The samples were prepared and analyzed according to the procedure for enrichment analysis, described in this manuscript. Then, to obtain the concentration, the amount of octanoate present in Liquigen^®^ is calculated from the observed ratio and the known amount of tracer added to the aliquot of Liquigen^®^, while taking into account naturally present background enrichments. Both the concentration of octanoate in Liquigen^®^ and the isotopic purity of the glyceryl tri[1,2,3,4-^13^C_4_] octanoate solution prepared by the pharmacy were found to be in accordance with the information provided on the certificates. For Liquigen^®^, we measured a concentration of 262.20 mg/mL, which deviated 3.7% from the concentration of 272.24 mg/mL in the product information. The enrichment of the fourfold labeled free fatty acid as analyzed by us was 97.3%, while the certificate of analysis from Sigma claimed 99.4% (deviating by 2.1%).

**Fig. 5:**
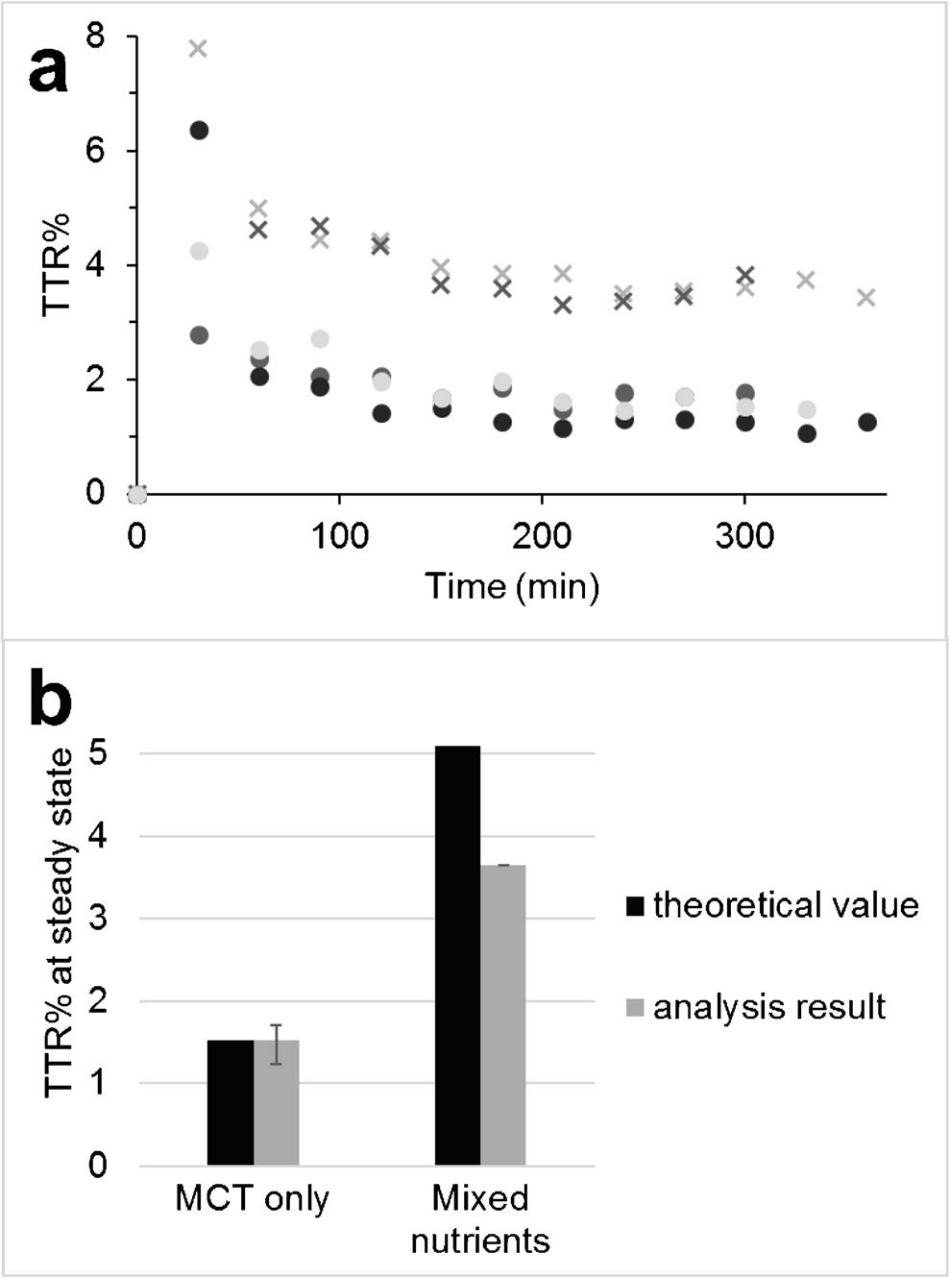
Analysis results obtained with the stable isotope protocol. **a** Enrichment results for 1,2,3,4-^13^C_4_-octanoate in plasma. Results obtained during MCT-only supplementation were marked by circles; the results obtained during the mixed nutrients supplementation were marked by crosses. Different shades were used to indicate different subjects. **b** Comparison between the average theoretical TTR% values of the different supplemental nutrition (calculated from the TTR% value of the administered glyceryl trioctanoate) and the analysis result (TTR% of 1,2,3,4-^13^C_4_-octanoate in plasma). The range of the data was represented by the error bars

The octanoate enrichment results obtained for the blood samples of the volunteers are presented in Fig. 5 and Table 1. Steady state was reached in each patient between 120 and 180 min after start of the infusion, as can be seen in Fig. 5. The isotopic enrichments during steady state are represented per patient in Table 1.

**Table 1:** Overview of octanoate enrichment; theoretical tracer-tracee ratio percent (TTR%) administered during the study protocol; the observed TTR% in plasma during steady state, reached at the latest after 180 min after start of the infusion; and the deviation of the analysis result in comparison with the theoretical TTR%

Supplemental nutrition	Theoretical enrichment (TTR%)	Average deviation of analysis results from theoretical value (%)	Individual analysis results (average during steady state) (TTR%)	SD of individual results during steady state	Deviation from theoretical value (%)
MCT only	1.53	0.2	1.71	0.12	12.1
			1.24	0.09	− 18.4
			1.63	0.19	6.7
Mixed nutrients	5.10	− 28.5	3.64	0.17	− 28.5
			3.65	0.35	− 28.5

## Discussion

We describe the development and validation of a new gas chromatography–mass spectrometry (GC-MS) method for the measurement of octanoate enrichment in plasma samples obtained after the administration of stable isotope–labeled MCT.

The most common derivatization method for analyzing fatty acids is methyl esterification [14]. For purposes of determining the amount of MCT that is elongated, fatty acid enrichments in the plasma samples obtained during the isotope infusion protocol were analyzed after methyl esterification by gas chromatography–combustion–isotope ratio mass spectrometry (GC-C-IRMS) [3, 13]. GC-C-IRMS is capable of obtaining very precise measurements of enrichments, especially at low enrichment levels as observed in this protocol [15]. Accurate analysis of octanoate enrichment in plasma by GC-C-IRMS was not possible after methyl esterification of the samples. Due to the relatively high enrichments of 1,2,3,4-^13^C_4_-octanoate and the low concentrations of octanoate in plasma, GC-MS is the instrument of choice [16].

However, during the use of GC-MS, fragmentation of the octanoate methyl ester was unfavorable. The mass spectrum of methyl octanoate (Fig. 3b) shows more abundant fragments in the lower *m*/*z* values, though these fragments have a low specificity for octanoate. Fragments with a low *m*/*z* value are generally unspecific, as they are representative for functional groups of the analyte or the derivatization reagent. There is a high probability that other compounds present in human plasma have fragments with these *m*/*z* values in their mass spectra. The more specific fragments (with higher *m*/*z* values) are not abundant enough for sensitive analysis. When analyzing samples from fasted patients, we observed a signal-to-noise ratio of < 10 in the obtained chromatogram. Moreover, we observed in vitro contamination of unlabeled octanoate following the methyl ester derivatization sample preparation. Furthermore, very careful handling of the samples was needed, as the methyl ester of octanoate is very volatile and was easily lost during sample handling [11]. Also, after derivatization with methanolic HCl, we observed elevated enrichment levels of non-enriched samples. We found that this observed (falsely high) enrichment after methyl esterification is inversely related to the peak area of the unlabeled octanoate peak: the lower this peak area, the higher the enrichment. Therefore, a new sample preparation method and a new GC-MS method were developed, which both is more sensitive and minimizes the risk of contamination.

*N*-tert-Butyldimethylsilyl-*N*-methyltrifluoroacetamide (MTBSTFA) was tested for the derivatization of octanoate [17]. The drawback of this derivatization reaction is the need for a separate saponification step, whereas with other reagents, transesterification (saponification and derivatization in a single step) is possible. Saponification through the method described by Csonka et al. [18] prior to MTBSTFA derivatization is possible, though a large contribution of unlabeled octanoate was observed in blank samples. MTBSTFA from other suppliers and modifications of the sample preparation procedure, such as reagent volume and reaction temperature, did not solve this problem.

Butylchloroformate was also investigated as a derivatization reagent and gave spectra corresponding to those in the NIST library for the derivatized free octanoate standard [19]. However, from our tests, it appeared that chloroformates are not capable of transesterification, not even at an elevated temperature (90 °C).

Isobutanol, butanol, and propanol with acetylchloride resulted in mass spectra that showed both specificity (higher mass fragments that were abundant) and complete transesterification. A drawback of this method is that due to the high miscibility of these alcohols with other solvents, it is not possible to extract and concentrate the samples. The concentration of the analyte—and, therefore, the analytical response (peak area)—is dependent on the volume of the reaction mixture in which derivatization was carried out. However, when the volume of the derivatization reagent added to the sample is too low, the recovery of the reaction is diminished.

Generally, fatty acids are extracted from plasma prior to derivatization, though it was demonstrated that direct extraction and derivatization in plasma gave a very similar fatty acid profile compared with extraction and subsequent derivatization [20]. In our experiments, the extraction of plasma prior to derivatization resulted in a higher contribution of contaminating octanoate observed in blank samples. Therefore, this step was omitted. It appeared that every additional handling of the samples increased the amount of contaminant. Thus, we decided to carry out the reaction directly in plasma. Simultaneously, the risk of contamination was reduced, as fewer handlings were needed in this new protocol.

Rinsing all glasswork prior to use caused a major decrease in the contribution of contamination to the blank samples. Isobutanol was shown to have the lowest amount of contamination compared with butanol and propanol. The elevated enrichment levels in non-enriched plasma samples, as observed in derivatization with methanolic HCl, did not present itself with isobutanol HCl derivatization. The volatility of the isobutyl octanoate is lower in comparison with that of methyl octanoate, decreasing the risk of losses during sample preparation. Moreover, methyl octanoate occurs as a natural product, adding to the uncertainty of the results, whereas isobutyl octanoate is not.

Because of the small molecule size, the possibilities for increasing the analytical performance are limited. Typically, analysis in multiple reaction monitoring (MRM) mode offers increased selectivity and sensitivity in comparison with analysis in single ion monitoring (SIM) mode. A triple quadrupole mass spectrometer was used to perform the analysis, to create the possibility of performing an MRM analysis. However, this turned out to not be advantageous for this derivate, as the potential precursor ions were already small and a potential product ion was not found. Therefore, in the eventual method, the triple quadrupole mass spectrometer was operated in the single ion monitoring (SIM) mode. This means that the validated analysis method can also be performed using a GC-MS with a single quadrupole instead of the GC tandem mass spectrometer (GC-MS/MS) used during these experiments.

Unfortunately, for the 1,2,3,4-^13^C_4_-octanoate isotope label, only the glyceryl tri[1,2,3,4-^13^C_4_] octanoate solution from the pharmacy (in water) was available. To prepare the calibration standards and control pools, we had to pipet small quantities of this solution. This ended up causing difficulties with repeatability, as the solution was not homogeneous enough at that level (e.g., 20 μL). To solve this problem, we extracted a larger volume of the aqueous solution with chloroform and methanol and, after drying it under nitrogen, redissolved the extracted glyceryl tri[1,2,3,4-^13^C_4_] octanoate in hexane. In this way, we prepared a stock solution in a more appropriate solvent and obtained a homogeneous solution. This improved the accuracy of pipetting small amounts, as observed during the preparation of standard curves.

We created control samples by adding glyceryl tri[1,2,3,4-^13^C_4_] octanoate directly to pool plasma. However, MCT appears in blood as free fatty acids, bound to albumin [2]. Our control samples do not identically reflect this physiological appearance, which may be why we found larger deviations in the reproducibility experiments than we typically observe during isotope studies, although the precision fell within the limits that were prespecified in the “Materials and methods” section.

As can be seen from Fig. 5, we found a steady state of octanoate enrichment in plasma samples obtained during the stable isotope protocol. Since the continuous infusion was preceded by a priming dose, the enrichment was elevated during the first period, and after 120–180 min, a steady state was reached in all subjects. We expected the enrichment for octanoate in plasma to correspond to the enrichment in the diet. The concentration of octanoate in plasma is severely influenced by the diet, and since the subjects started the test in the fasted state, we expected that all circulating octanoate was originating from the diet. If this is the case, the dietary enrichment and plasma enrichment should agree. The analysis results for the octanoate enrichment in plasma (Fig. 5 and Table 1) were in agreement with the expected enrichment within biological variation (deviation < 20%) except for the mixed nutrients day (the deviations found were − 28.5%). The discrepancy between the enrichment in the diet and plasma enrichment for octanoate was also described by Carnielli et al. [3], who observed a deviation of even − 90%. Their paper describes several sources of unlabeled octanoate, including lipogenesis from glucose [3]. In our study, the deviating results were obtained during the mixed nutrients day, when MCT was provided in combination with proteins and carbohydrates. Following our results, lipogenesis from glucose as a source of unlabeled octanoate could be an explanation for these results, as the significant deviation is observed on the mixed nutrients day and not on the MCT-only day.

## Conclusion

We developed a method for accurately analyzing 1,2,3,4-^13^C_4_-octanoate enrichment in plasma samples through transesterification with acetyl chloride in isobutanol. We found that this new derivatization procedure increases sensitivity by a factor of 20 and is more accurate compared with previously described methods. With this method, we were able to analyze the octanoate enrichment in plasma samples obtained during a stable isotope infusion protocol that will be applied to patients with long-chain fatty acid oxidation disorder, to determine the metabolic fate of orally administered MCT.

## Data Availability

The datasets generated during and/or analyzed during the current study are available from the corresponding author on reasonable request.
